# Toxoplasma Co-infection Prevents Th2 Differentiation and Leads to a Helminth-Specific Th1 Response

**DOI:** 10.3389/fcimb.2017.00341

**Published:** 2017-07-25

**Authors:** Norus Ahmed, Timothy French, Sebastian Rausch, Anja Kühl, Katrin Hemminger, Ildiko R. Dunay, Svenja Steinfelder, Susanne Hartmann

**Affiliations:** ^1^Department of Veterinary Medicine, Institute of Immunology, Freie Universität Berlin Berlin, Germany; ^2^Institute of Inflammation and Neurodegeneration, Otto-von-Guericke University Magdeburg, Germany; ^3^Division of Gastroenterology, Medical Department, Infection and Rheumatology, Research Center ImmunoSciences Berlin, Germany

**Keywords:** Th2, Th1, *Toxoplasma gondii*, *Heligmosomoides polygyrus*, co-infection, helminth

## Abstract

Nematode infections, in particular gastrointestinal nematodes, are widespread and co-infections with other parasites and pathogens are frequently encountered in humans and animals. To decipher the immunological effects of a widespread protozoan infection on the anti-helminth immune response we studied a co-infection with the enteric nematode *Heligmosomoides polygyrus* in mice previously infected with *Toxoplasma gondii*. Protective immune responses against nematodes are dependent on parasite-specific Th2 responses associated with IL-4, IL-5, IL-13, IgE, and IgG1 antibodies. In contrast, *Toxoplasma gondii* infection elicits a strong and protective Th1 immune response characterized by IFN-γ, IL-12, and IgG2a antibodies. Co-infected animals displayed significantly higher worm fecundity although worm burden remained unchanged. In line with this, the Th2 response to *H. polygyrus* in co-infected animals showed a profound reduction of IL-4, IL-5, IL-13, and GATA-3 expressing T cells. Co-infection also resulted in the lack of eosinophilia and reduced expression of the Th2 effector molecule RELM-β in intestinal tissue. In contrast, the Th1 response to the protozoan parasite was not diminished and parasitemia of *T. gondii* was unaffected by concurrent helminth infection. Importantly, *H. polygyrus* specific restimulation of splenocytes revealed *H. polygyrus*-reactive CD4^+^ T cells that produce a significant amount of IFN-γ in co-infected animals. This was not observed in animals infected with the nematode alone. Increased levels of *H. polygyrus*-specific IgG2a antibodies in co-infected mice mirrored this finding. This study suggests that polarization rather than priming of naive CD4^+^ T cells is disturbed in mice previously infected with *T. gondii*. In conclusion, a previous *T. gondii* infection limits a helminth-specific Th2 immune response while promoting a shift toward a Th1-type immune response.

## Introduction

Gastrointestinal nematode infections affect around 24% of the human population (WHO, 2017 last modified January, 2017 http://www.who.int/mediacentre/factsheets/fs366/en/; Horton, 2003). These parasites are not necessarily fatal though they cause high morbidity including malnourishment, intestinal inflammation and anemia in both acutely and chronically infected patients. In particular, school children are affected hindering their mental and physical development. Moreover, individuals living in endemic areas get frequently re-infected and immunity only develops after several decades (Mosmann and Coffman, 1989). Most areas endemic for nematodes are co-endemic for various diseases, such as malaria, tuberculosis, toxoplasmosis (Bahia-Oliveira et al., 2009), leishmaniasis and salmonellosis (Hotez and Kamath, 2009). Every pathogen should be encountered by a tailored immune response engaging a certain set of effector molecules, however this is a problem for the immune system during co-infections. Seroprevalence of *Toxoplasma gondii* reaches up to 70% in certain areas with high chances of co-infection with different pathogens among the human population (Bahia-Oliveira et al., 2009; Pappas et al., 2009). High prevalence of *T. gondii* is found in tropical regions, such as Latin America, Middle East, Africa and Southeast Asia. On the other hand *T. gondii* infection evokes and is controlled by a very different immune response compared to helminth infections. Detailed experimental studies are required to unravel how acute or chronic infections with certain pathogens affect the immune system, when faced with new challenges by unrelated infections.

Helminth infections are typically characterized by the activation and expansion of CD4^+^ T helper 2 (Th2) cells, which express the transcription factor GATA-3 and secrete interleukin (IL)-4, IL-5, IL-9, and IL-13, leading to IgG1 and IgE antibody production. In addition, the Th2 response leads to eosinophilia, enhanced mucus production, specific granuloma-formation around larvae, as well as specific priming of innate cells, such as macrophages. This immune response can directly, or via upregulation of effector molecules, such as RELM-β produced by goblet cells, reduce worm fecundity and enhance parasite expulsion (Artis et al., 2004; Owyang et al., 2006).

Helminths evade the host immune responses due to host-parasite interactions (McSorley and Maizels, 2012) whereby the Th2 immune responses are actively regulated by the worm (Yazdanbakhsh et al., 2001). Thus, the Th2 response is accompanied by the emergence of parasite-induced regulatory cells, such as regulatory B-cells (Breg) (Hussaarts et al., 2011), regulatory T-cells (Treg) (Taylor et al., 2012) and alternatively activated macrophages (AAM) (Gordon and Martinez, 2010), which are known to limit parasite-specific and unspecific immune responses (Steinfelder et al., 2016). *Heligmosomoides polygyrus* is a well-studied strictly intestinal helminth of mice featuring all of the aforementioned characteristics (Reynolds et al., 2012). In addition to this, it is a widespread natural infection of wild mice (Maaz et al., 2016). The parasites are orally taken up as infective stage 3 larvae (L3), which subsequently embed themselves into the small intestinal wall to develop into L4. They then emerge as adults into the lumen, where they prevail for weeks before being expelled, depending on the mouse strain (Bansemir and Sukhdeo, 1994; Reynolds et al., 2012).

In contrast, *T. gondii* is an obligate intracellular protozoan parasite, which orally infects warm-blooded vertebrate hosts. After an initial intestinal phase, *T. gondii* spreads systemically and converts into a dormant stage in muscle and brain tissues (Dubey, 2008). Human infections with *T. gondii* are common and are mostly asymptomatic in immunocompetent individuals (Ho-Yen and Joss, 1992), although they may trigger basal inflammation (Parlog et al., 2015). Here, IFN-γ plays an important role in the containment of *T. gondii* (Ely et al., 1999). However, a previous latent infection in immunocompromised humans can reactivate and cause life threating encephalitis if left untreated (Luft et al., 1984; Montoya and Liesenfeld, 2004). During *T. gondii* infection a T helper 1 (Th1) immune response is elicited. This provides a strong, protective immune response and is characterized by dendritic cells (DC) producing IL-12. The production of IL-12 leads to the differentiation of CD4^+^ T cells into Th1 cells expressing the transcription factor T-bet and the secretion of IFN-γ. Additionally, innate cells, such as neutrophils, NK-cells and innate lymphoid cells provide other early sources of IFN-γ (Gazzinelli et al., 1994; Sturge et al., 2013; Klose et al., 2014).

Differentiation of Th1 and Th2 cells has been well documented in the literature. *In vitro*-based studies have shown that Th1 or Th2 polarizing conditions cause differentiated cells to lose their ability to completely switch phenotype after increased cell division (Murphy et al., 1996; Grogan et al., 2001). However, other studies have shown that Th subsets have the flexibility to produce non-lineage-specific cytokines (Murphy and Stockinger, 2010; O'Garra et al., 2011; Coomes et al., 2013). Furthermore, we have previously reported a subset of Th hybrid cells expressing both transcription factors T-bet and GATA-3, as well as producing IFN-γ and IL-4 at intermediate levels during helminth infections (Peine et al., 2013).

Studies on *H. polygyrus* and *T. gondii* co-infection in mice have so far focused on a previous infection with helminths and have shown that initially CD4^+^ and CD8^+^ T cell immunity against *T. gondii* is suppressed in mice. At later stages the *T. gondii*-specific CD4^+^ T cell response recovers whereas the CD8^+^ response remains disrupted (Khan et al., 2008). In line with this, other studies have shown that prior infection with *H. polygyrus* induced suppression of IL-12 dependent differentiation of effector CD8^+^ T cells as well as IFN-γ production against *T. gondii*. Interestingly, IL-4 and IL-10 deficiency was necessary to reverse the obstructing effect of *H. polygyrus* infection on the CD8^+^ T cell response toward *Toxoplasma* (Marple et al., 2017). The majority of co-infection studies despite being protozoan, viral or bacterial infection, have focused on infections with helminth first due to their ability to downmodulate immune responses (Rousseau et al., 1997; Liesenfeld et al., 2004; Chen et al., 2005, 2006; Graham et al., 2005; Su et al., 2005, 2014a,b; Weng et al., 2007; Khan et al., 2008; Noland et al., 2008; Miller et al., 2009; Frantz et al., 2010; Dias et al., 2011; Potian et al., 2011; Kolbaum et al., 2012; du Plessis et al., 2013; Osborne et al., 2014; Budischak et al., 2015; Coomes et al., 2015; Gondorf et al., 2015; Rafi et al., 2015; Obieglo et al., 2016). In light of the fact, that Th2 immunity against helminths is an ongoing challenge in humans and livestock, we aimed to investigate how a previous protozoan infection affects the development of Th2 responses in CD4^+^ T cells and protection against helminths.

We observed that a previous *T. gondii* infection leads to an overall suppression of *H. polygyrus*-specific Th2 immunity and enables *H. polygyrus*-specific CD4^+^ T cells to produce IFN-γ.

## Materials and methods

### Animals

Female NMRI and C57BL/6 mice (8 weeks old; purchased from Janvier, Saint Berthevin, France) were bred under specific pathogen-free (SPF) conditions at the Institute of Medical Microbiology, Universitätsklinikum Magdeburg, Germany or at the Institute of Immunology, Department of Veterinary Medicine, Freie Universität Berlin. The experiments performed followed the National Animal Protection Guidelines and were approved by the German Animal Ethics Committee for the protection of animals.

### Isolation of *T. gondii* tissue cysts and oral *T. gondii* infection

Female NMRI mice were infected orally (p.o.) with type II ME49 strain *T. gondii* cysts. After 8–10 months, tissue cysts were collected from the brains of chronically infected mice. After perfusion, brains were mechanically homogenized in 1 mL sterile PBS. Cysts were quantified using a light microscope and 8–10 weeks old female C57BL/6 mice were infected with 2-tissue cysts p.o. by oral gavage in a total volume of 200 μl/mouse (Möhle et al., 2016)

### *H. polygyrus* infection

The parasite *Heligmosomoides polygyrus* was retained by serial passage in C57BL/6 mice as described previously (Rausch et al., 2008). Mice aged 8–10 weeks old were infected by oral gavage with 200 L3 larvae in drinking water. On day 14 post infection (p.i.) mice were sacrificed by isofluorane inhalation.

### Detection of *T. gondii* parasitemia by PCR

*Toxoplasma gondii* burden was determined using Roche FastStart Essential DNA Green Master kit with manufacturer's protocol. TgB1 (TIBMolbiol, Berlin, Germany) was used as a target gene and Mm.ASL (TIBMolbiol, Berlin, Germany) as a reference (Heimesaat et al., 2014). Target/reference ratios were all calculated using the LightCycler® 480 Software release 1.5.0 (Roche, Germany).

### Worm fecundity and worm burden

Adult worms were isolated from the small intestine and counted. Female worms were subsequently kept individually (8 per mouse) in a 96 well round-bottom plate containing RPMI, 200 U/ml penicillin and 200 μg/ml streptomycin (all from PAA, Austria) at 37°C. After 24 h female *H. polygyrus* adults were removed from the wells and fecundity was determined by counting the eggs shed per female worm using a binocular microscope.

### Preparation of parasite antigen

*Heligmosomoides polygyrus* antigen (HpAg) was prepared from adults worms that were kept in culture containing 100 U/ml penicillin and 100 μg/ml streptomycin for 24 h as described before (Rausch et al., 2008).

### Cell culture

Cells were cultured in complete RPMI 1640 medium (cRPMI) containing 10% FCS 200 U/ml penicillin, 200 μg/ml streptomycin (all from PAA, Austria) in an incubator at 37°C and 5% CO_2_.

### Single cell suspension preparation

Spleen and mesenteric lymph nodes (mLN) were isolated from mice, homogenized and filtered through 70 μm cell strainers (BD Bioscience, San Jose, CA) to obtain single cell suspensions. The cells were then washed and re-suspended in cRPMI. Cells were counted using a CASY automated cell counter (Roche-Innovatis, Reutlingen, Germany). Small intestinal lamina propria (siLP) and epithelium (siE) cells were isolated by the removal of the whole small intestine that was then stored on ice in cold HBSS (w/o Ca^2+^ Mg^2+^) (PAA, Pasching, Austria) containing 2% FCS and 10 mM HEPES (PAA, Pasching, Austria). Small intestines were washed through with 20 ml cold buffer using a 20G needle. After washing, mesenteric fat and Peyer's patches were removed. The small intestines were then cut open longitudinally and mucus scraped off with forceps. Additionally adult *H. polygyrus* worms were removed and counted using forceps. Small intestines were then washed in HBSS/FCS/HEPES and cut in 1 cm pieces and stored in 20 ml HBSS/FCS/HEPES containing 0.154 mg/ml DTE (Sigma-Aldrich, St. Louis, MO). The 1 cm pieces were incubated in a tube shaker water bath (200 rpm, 37°C) for 15 min. This step was repeated twice and then the intestinal pieces were transferred into 20 ml HBSS/FCS/HEPES containing 5 mM EDTA and agitated at room temperature for 15 min, repeated three times. Intestinal pieces were put into fresh 20 ml HBSS/FCS/HEPES and the cell suspension containing epithelium and intraepithelial lymphocytes retrieved for density gradient isolation. Intestinal pieces were washed in RPMI to remove residual EDTA and then placed in 10 ml 37°C complete RPMI 1640 medium containing 0.1 mg/ml Liberase (Roche, Basel, Switzerland) and 0.1 mg/ml DNAse (Sigma-Aldrich, St. Louis, MO, USA). Intestinal pieces were then incubated at 37°C, 200 rpm for 30 min. After incubation, tubes containing intestinal pieces were vortexed vigorously to disturb remaining intestinal pieces. The intestinal pieces were then forced up and down through an 18G needle. Suspensions were then filtered over a 70 μm cell strainer and washed twice with HBSS/HEPES. The cell suspensions from siLP and siE were added on a percoll gradient (GE healthcare life sciences, Sweden). Lamina propria and epithelial cells were collected from the 40%/70% interface after centrifugation. Cells were washed in cRPMI and counted using Neubauer chambers (C-Chip, Biochrom GmbH, Berlin, Germany).

### Generation and antigen loading of bone marrow-derived dendritic cells

Naïve female C57BL/6 mice were used for isolation of bone marrow from the femur and tibia. Bone marrow cells were washed in RPMI medium and 1 × 10^6^ cells/ml were cultured in 10 ml/petri dish for 6 days in cRPMI containing 10 ng/ml recombinant murine GM-CSF (PeproTech, Hamburg, Germany). On day 3, 10 ml of cRPMI with 10 ng/ml GM-CSF was added. On day 6 BmDC were counted, seeded at 1 × 10^5^ cells/well in a 96 well plate and stimulated with 50 μg/ml of *H. polygyrus* antigen (HpAg) for 24 h. Cells were then washed and used for the co-culture experiment.

### Antigen-specific restimulation of CD4^+^ splenocytes

1 × 10^6^ splenocytes were co-cultured with 1 × 10^5^ BmDC pulsed overnight with HpAg for 5 h in the presence of 3 μg/ml Brefeldin A (eBioscience, San Diego, CA, USA) followed by intracellular staining for CD154 and cytokines.

### Cell proliferation assay

Spleen cells were isolated and stained with CFSE **(**Carboxyfluorescein succinimidyl ester**)**. 1 × 10^6^ CFSE labeled cells were cultured with 20 μg/ml *H. polygyrus* antigen. Cells were cultured for 6 days and restimulated with 1 μg/ml PMA and 1 μg/ml Ionomycin (both Sigma-Aldrich, St. Louis, MO, USA) in the presence of 3 μg/ml Brefeldin A (eBioscience, San Diego, CA, USA) followed by intracellular staining.

### Realtime PCR

RNA was isolated from intestinal tissue sections previously stored at −80°C via homogenization in RNA lysis buffer. The tissue supernatant was processed with a innuPREP RNA kit (Analytik Jena, Jena, Germany) following manufacturer's instructions. 2 μg of RNA was reverse transcribed to cDNA using the High Capacity RNA to cDNA kit (Applied Biosystems, Foster City, CA). The relative expression of β-actin, resistin-like molecule-beta (Relm-β), IL-12 and IFN-γ, was determined by Real Time PCR using 10 ng of cDNA and the FastStart Universal SYBR Green Master Mix (Roche, Basel, Switzerland). Primer pairs used for gene amplification were as follows: β-actin forward: GGCTGTATTCCCCTCCATCG, reverse: CCAGTTGGTAACAATGCCATGT, Relm-β (Retnlb) forward: GGCTGTGGATCGTGGGATAT, reverse: GAGGCCCAGTCCATGACTGA. IL-12 forward: ATGGCCATGTGGGAGCTGGAGAAAG, reverse: GTGGAGCAGCAGATGTGAGTGGCT. IFN-γ forward: ATgAACgCTACACACTgCATC, reverse: CCATCCTTTTgCCAgTTCCTC. Primer pair efficiency was determined via a standard curve. The mRNA expression was normalized to the β-actin housekeeping gene and calculated by Roche Light Cycler 480 software.

### Cytokine detection by ELISA

3.5 × 10^5^ splenocytes were stimulated with 20 μg/ml *H. polygyrus* antigen or 2 μg/ml anti-CD3 and anti-CD28 (both eBioscience, San Diego, CA, USA) for 6 days. Supernatants were analyzed for IL-4, and IFN-γ using Ready-Set-Go Elisa Kits (eBioscience, San Diego, CA, USA) according to the manufacturer's instructions.

### Antibody isotype detection by ELISA

*Heligmosomoides polygyrus-*specific IgG1 and IgG2a were measured in serum. 96 well microtiter plates coated with 10 μg/ml *H. polygyrus* antigen were incubated with serum diluted 1:100 with 3% BSA in PBS. Bound antibody isotypes were detected using alkaline phosphatase conjugated anti-mouse IgG1 and IgG2a antibodies diluted 1:5000 each (Rockland, PA, USA) and para-nitrophenylphosphate (Sigma, Steinheim am Albuch, Germany). All samples were run in duplicates. Arbitrary units were calculated using pooled samples as reference.

### Flow cytometry

For surface and intracellular staining, the monoclonal antibodies listed were used: CD4 (PerCP) (RM4-5); CD192 (CCR2) (Alexa 647) (SA203G11) all from BioLegend (Biozol); CD8 (53-6.7); IL-4 (PE-Cy7) (11B11); IL-5 (PE) (TRFK5); IL-13 (Alexa 488) (eBio13A); IFN-γ (eFluor 450) (XMG1.2); CD154 (PE) (MR1); Foxp3 Alexa 488 (FJK-16s); GATA3 (eFluor 660) (TWAJ); Dead Cell Exclusion Marker (DCE) (efluor 780); DCE (efluor 506); Siglec F (PE) (E50-2440); T-bet (PE) (eBio4B10); IL-13 (eFluor 660) (eBio13A); CD11b (PE) (M1/70); F4/80 (PerCP-Cy5.5) (BM8); Ly-6G (Gr-1) (PE-Cy7) (RB6-8c5); Ly-6C (eFluor 450) (HK1.4); TNF-α (Alexa488) (MP6-XT22) all from eBioscience, San Diego, CA, USA.

For intracellular staining of cytokines and transcription factors cells were fixed and permeabilized using the fix/perm buffer kit (eBioscience, San Diego, CA, USA). FACSCantoII flow cytometer and FACSAriaIII sorter (both BD Bioscience, Heidelberg, Germany) were used for cell analysis. FlowJo software 10.2 was used for final analysis (Tree star Inc., Ashland, OR, USA).

### Statistics

Experiments were performed as shown and displayed as mean ± SD or mean ± SEM as indicated. Statistical analysis was performed using GraphPad Prism software (La Jolla, CA, USA). The level of significance was determined using the Mann Whitney *U*-test or Kruskal-Wallis with Dunn's multiple comparison test.

## Results

### Prior infection with *T. gondii* results in increased fecundity of *H. polygyrus* in co-infection

**Figure 1:**
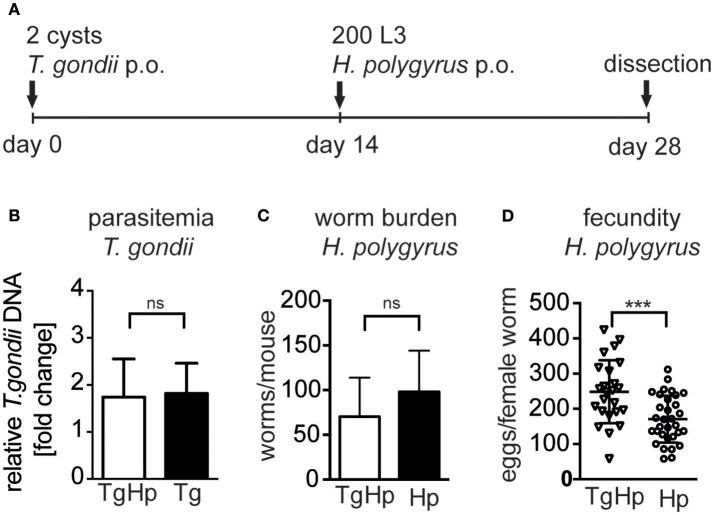
Experimental design **(A)**, C57BL/6 mice were infected with 2 ME49 *T. gondii* tissue cysts and/or 200 *H. polygyrus* L3 larvae. **(B)** Parasitemia of *T. gondii* in the heart, pooled from two independent experiments, fold change compared to uninfected, data shown as mean ± SEM, *n* = 6–7 **(C)** *H. polygyrus* worm burden. **(D)** Fecundity of female worms. Panels **(C,D)** are representative of three independent experiments with *n* = 4. Data shown as mean ± SD; statistical analysis was performed using the Mann-Whitney test, ^***^P ≤ 0.001.

We investigated whether infection with *T. gondii* affects the control of helminth parasites in the small intestine (Figure 1A). Mice infected with *T. gondii* for 14 days followed by *H. polygyrus* infection did not show altered parasitemia of *T. gondii* in the heart compared to mice infected with *T. gondii* alone (Figure 1B). Similarly, co-infected mice did not show a significant difference in *H. polygyrus* adult worm burden compared to *H. polygyrus* single infection (Figure 1C). However, female worms retrieved from co-infected mice showed a significantly higher fecundity compared to worms from *H. polygyrus* single infection (Figure 1D). Thus, a previous and on-going infection with the protozoan parasite *T. gondii* leads to a decline of anti-helminthic control in terms of fecundity leading to enhanced egg production.

### Previous infection with *T. gondii* selectively restricts Th2 polarization in response to helminth infection

**Figure 2:**
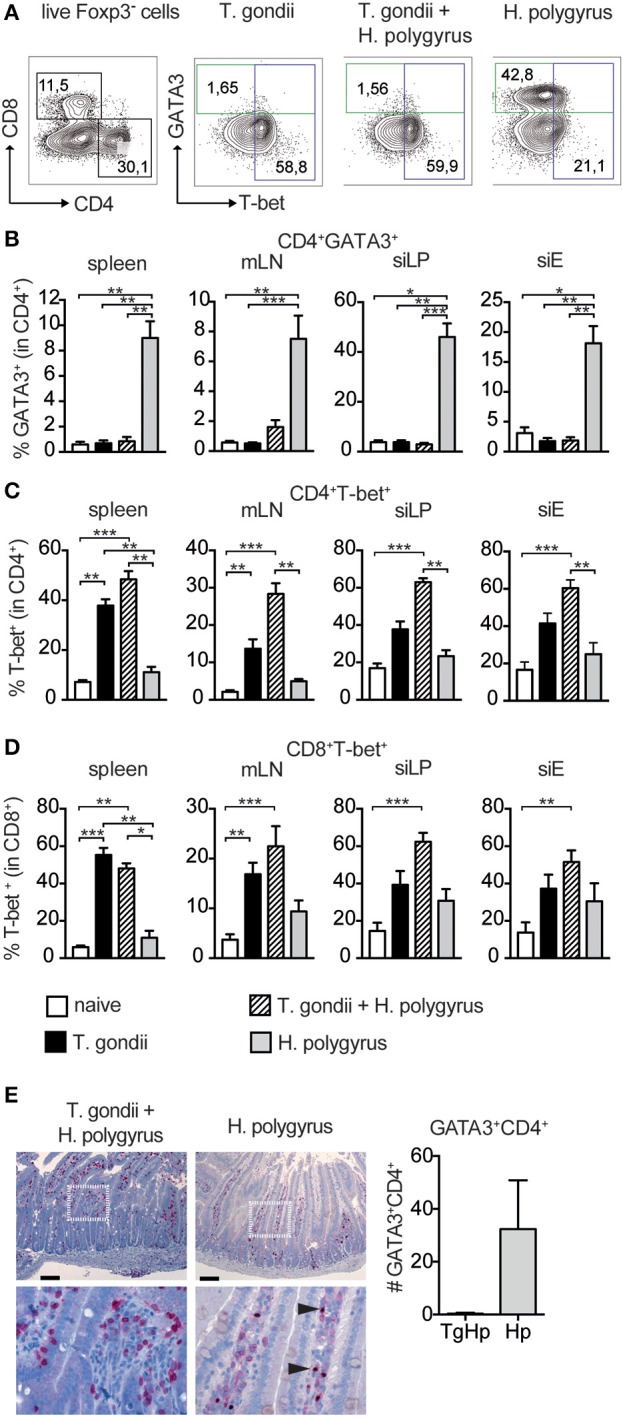
Restricted Th2 responses in co-infected mice. Cells from spleen, mesenteric lymph nodes (mLN), small intestinal lamina propria (siLP) and small intestinal epithelium (siE) were isolated and stimulated with PMA and ionomycin in the presence of Brefeldin A followed by intranuclear staining for the lineage transcription factors GATA3 and T-bet. Gating strategy shown in **(A)**, Bar graphs showing frequencies of CD4^+^ T cells expressing GATA3 **(B)**, T-bet **(C)**, and T-bet expression in CD8^+^ T cells **(D)**. GATA3 expression in the duodeunum of the small intestine with scale bar of 100 μm **(E)**, data shown as mean ± SEM pooled from 2 independent experiments *n* = 6, statistical analysis was performed using the Mann-Whitney test. **(B–D)** shown as mean ± SEM, pooled from two independent experiments with *n* = 8–10. Statistical analysis was performed using the Kruskal-Wallis with Dunn's multiple comparison test, ^*^P ≤ 0.05, ^**^P ≤ 0.01, and ^***^P ≤ 0.001.

The increased fecundity in co-infected mice, described as number of eggs produced per female worm *ex vivo*, might be due to insufficient Th2 immune pressure. To test this we compared systemic and local immune responses in co-infected and single infected groups. To differentiate between the contrasting immune responses the Th2-lineage marker GATA3 (Zheng and Flavell, 1997) and the Th1-lineage marker T-bet (Szabo et al., 2000) were used. GATA3 is also present on a subset of regulatory T cells that also express Foxp3. Regulatory T cells were excluded using their expression of Foxp3 (Figure 2A; Wohlfert et al., 2011).

The frequency of GATA3^+^Foxp3^−^CD4^+^ was drastically reduced in the spleen, small intestinal lamina propria (siLP) and small intestinal epithelial layer (siE) in co-infected mice compared to mice infected with *H. polygyrus* only. The reduction in GATA3 was similar to levels found in uninfected mice (Figure 2B). Furthermore, histology of the small intestine of co-infected mice showed no GATA-3 expression compared to *H. polygyrus* infected mice (Figure 2E). On the contrary, the Th1-lineage marker T-bet in CD4^+^ T cells was expressed in co-infected mice with higher levels to *T. gondii* alone in all compartments (Figure 2C). In addition to this, T-bet^+^ expression in CD8^+^ T cells was higher in siLP, siEL and mLN in co-infected mice compared to mice infected with *T. gondii* alone (Figure 2D).

### Co-infection leads to suppression of Th2 cytokine responses in CD4^+^ T cells

**Figure 3:**
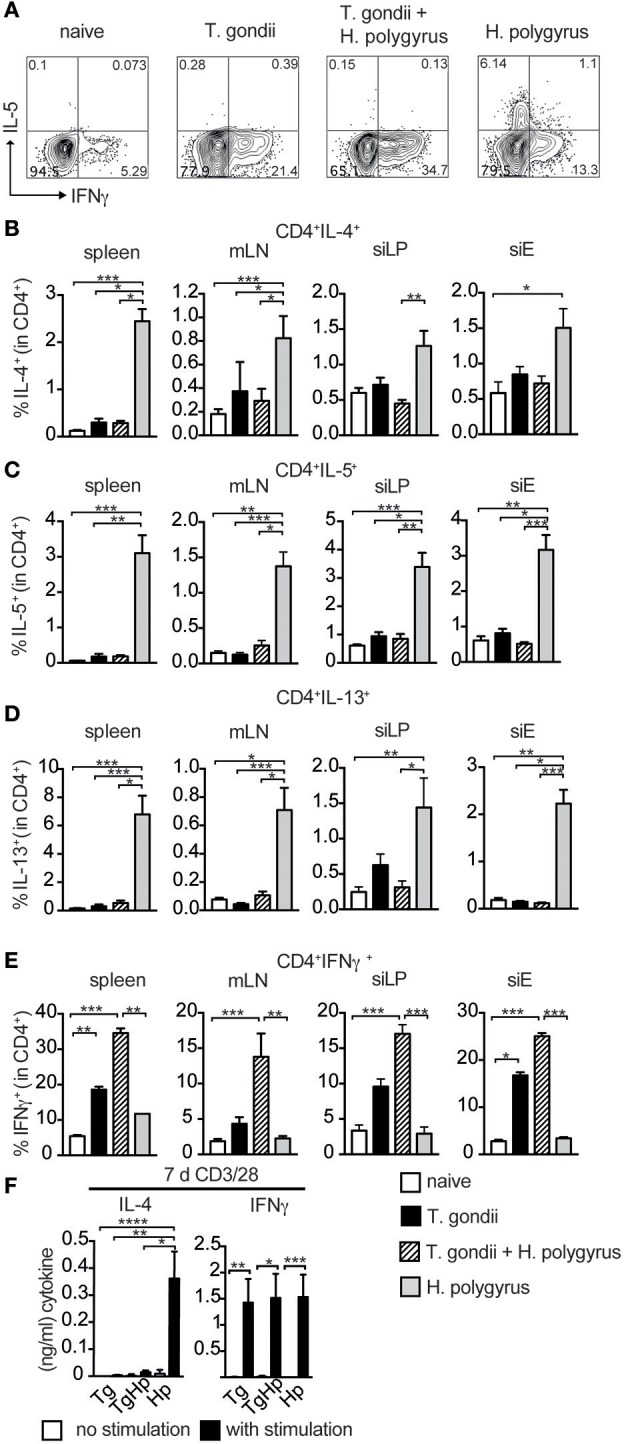
Th2 but not Th1 immune responses are absent in co-infected mice. Cells from spleen, mLN, siLP, and siE of single and co-infected animals were isolated and stimulated with PMA and ionomycin in the presence of Brefeldin A followed by intracellular cytokine staining. Gating strategy for the cytokines IL-5 and IFN-γ in CD4^+^ cells isolated from spleen **(A)**. Bar graphs showing frequencies of CD4^+^ T cells expressing IL-4 **(B)**, IL-5 **(C)**, IL-13 **(D)**, and IFN-γ **(E)** in spleen, mLN, siLP, and siE. **(F)** IL-4 and IFN-γ production detected by ELISA in supernatant from 3 × 10^5^ splenocytes stimulated with (black bars) and without (white bars) anti-CD3/CD28 antibodies. **(B–F)** Data shown as mean ± SEM, pooled from two independent experiments with *n* = 8–9 Statistical analysis was performed using the Kruskal-Wallis with Dunn's multiple comparison test, ^*^P ≤ 0.05, ^**^P ≤ 0.01, and ^***^P ≤ 0.001.

The lack of GATA3 expression prompted us to investigate whether the failure to differentiate into Th2 cells (Figure 2) extends to the inability to secrete Th2 cytokines. CD4^+^ T cells were restimulated with PMA/Ionomycin and cytokine expression was assessed (Figure 3A). The frequency of CD4^+^ cells producing IL-4 in spleen, siLP and mLN showed a marked reduction in co-infected mice compared to the *H. polygyrus* single-infected group. However, only a trend in the reduction of IL-4 was observed in siEL (Figure 3B). Also, the frequency of IL-5 and IL-13 in spleen, siLP, siEL, and mLN showed a significant reduction in co-infected mice in comparison to *H. polygyrus* infection alone (Figures 3C,D). This observation shows that the Th2 immune responses are suppressed locally as well as systemically in mice previously infected with *T. gondii*. On the contrary, CD4^+^ cells producing the Th1 cytokine IFN-γ showed an increase in the co-infected group compared to mice infected with *T. gondii* alone (Figure 3E), mirroring the enhanced T-bet expression found in the same cell population (Figure 2C). Similarly, T-bet and IFN-γ expression in CD8^+^ T cells were comparable in mice single and co-infected with *T. gondii* (Figure S1). To further investigate this finding, splenocytes were stimulated with anti-CD3/28 to evaluate the ability of these cells to produce the Th2 cytokine IL-4 (Figure 3F). The co-infected group did not show any IL-4 production compared to the *H. polygyrus*-infected group whereas IFN-γ was produced in all groups.

**Figure 4:**
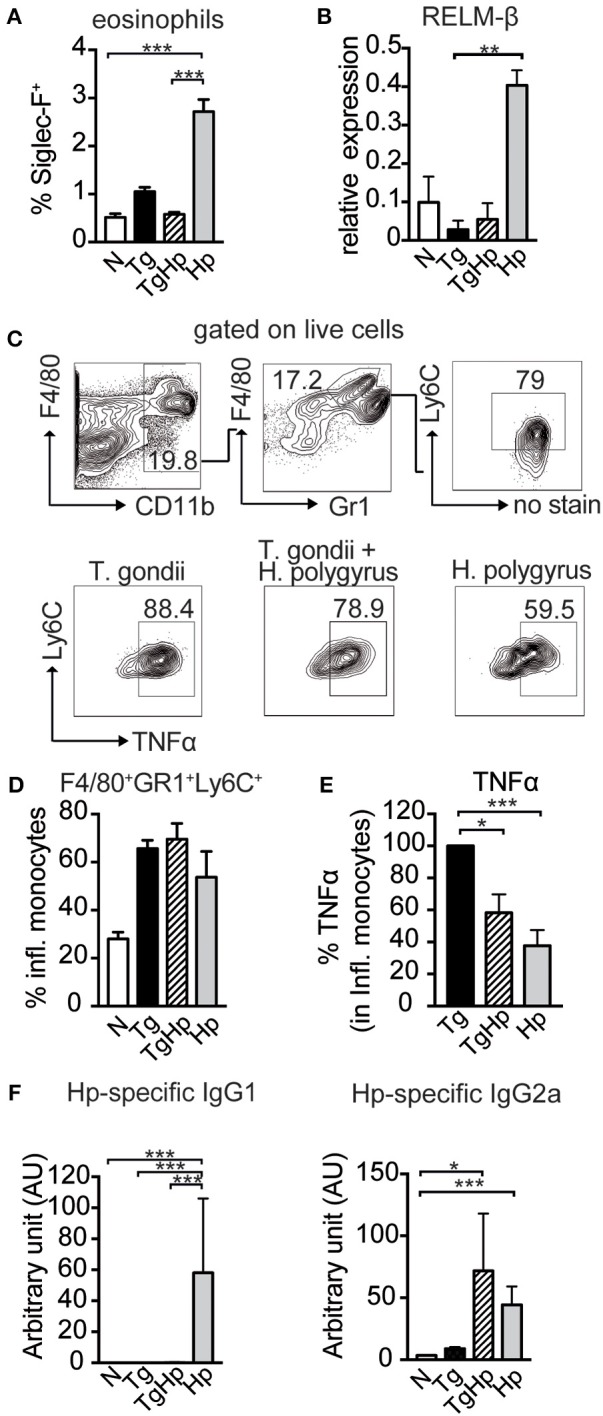
Impact of co-infection on Th1- and Th2-dependent effector cells, effector molecules and antibody isotypes. **(A)** Bar graph showing the frequency of Siglec-F^+^ eosinophils in the spleen, pooled from 2 independent experiments *n* = 7–8. **(B)** Relative Gene Expression of RELM-β compared to housekeeping gene β-actin (pooled from two experiments, *n* = 5–8). **(C,D)** Gating strategy and bar graphs showing frequency of inflammatory monocytes (F4/80^+^GR1^+^Ly6C^+^) in spleen stimulated with LPS (pooled from two experiments, *n* = 8). **(E)** Percentage of TNFα, with the response of the *T. gondii* infected group represented as 100%, stimulated with LPS, pooled from two experiments, *n* = 9. **(F)** *H. polygyrus* specific IgG1 and IgG2a detected by ELISA, *n* = 7–8 pooled from two independent experiments. Data shown as mean ± SEM. Statistical analysis was performed using the Kruskal-Wallis with Dunn's multiple comparison test, ^*^P ≤ 0.05, ^**^P ≤ 0.01, and ^***^P ≤ 0.001.

Next, we evaluated effector mechanisms that are downstream of a Th2 response. As the appearance of Th2 cytokines in helminth infection is associated with eosinophilia, we tested the influence of a co-infection on the number of eosinophil granulocytes. Consistent with the lack of Th2 cytokine secretion (Figure 3), co-infected mice showed a significant reduction in the frequency of eosinophils in spleen (Figure 4A) compared to *H. polygyrus* single infection. Similarly, in mice infected with both *T. gondii* and *H. polygyrus* there was reduction in RELM-β expression in small intestinal tissue compared to *H. polygyrus* single infection (Figure 4B). This indicates that effector cells and molecules normally elicited by helminth infection are directly affected by the absence of Th2 cytokines in a co-infection setting. In contrast, no changes were observed in the frequency of inflammatory monocytes (F4/80^+^GR1^+^Ly6C^+^) in both single and co-infected mice (Figures 4C,D). However, a significant reduction was observed in the production of TNF-α in inflammatory monocytes of co-infected mice in comparison to *T. gondii* single infected mice (Figure 4E),. In addition to this, co-infected mice showed a drastic reduction in *H. polygyrus* specific IgG1 compared to *H. polygyrus* single infection, which showed a prominent parasite-specific IgG1 response (Figure 4F). In contrast, helminth-specific IgG2a is increased in co-infected mice compared to mice infected with helminths alone. Thus, this data indicates that effector cells, effector molecules and the respective antibody response normally elicited by helminth infections are drastically altered in mice previously infected with *T. gondii*.

### Co-infection results in a helminth-specific Th1 profile

**Figure 5:**
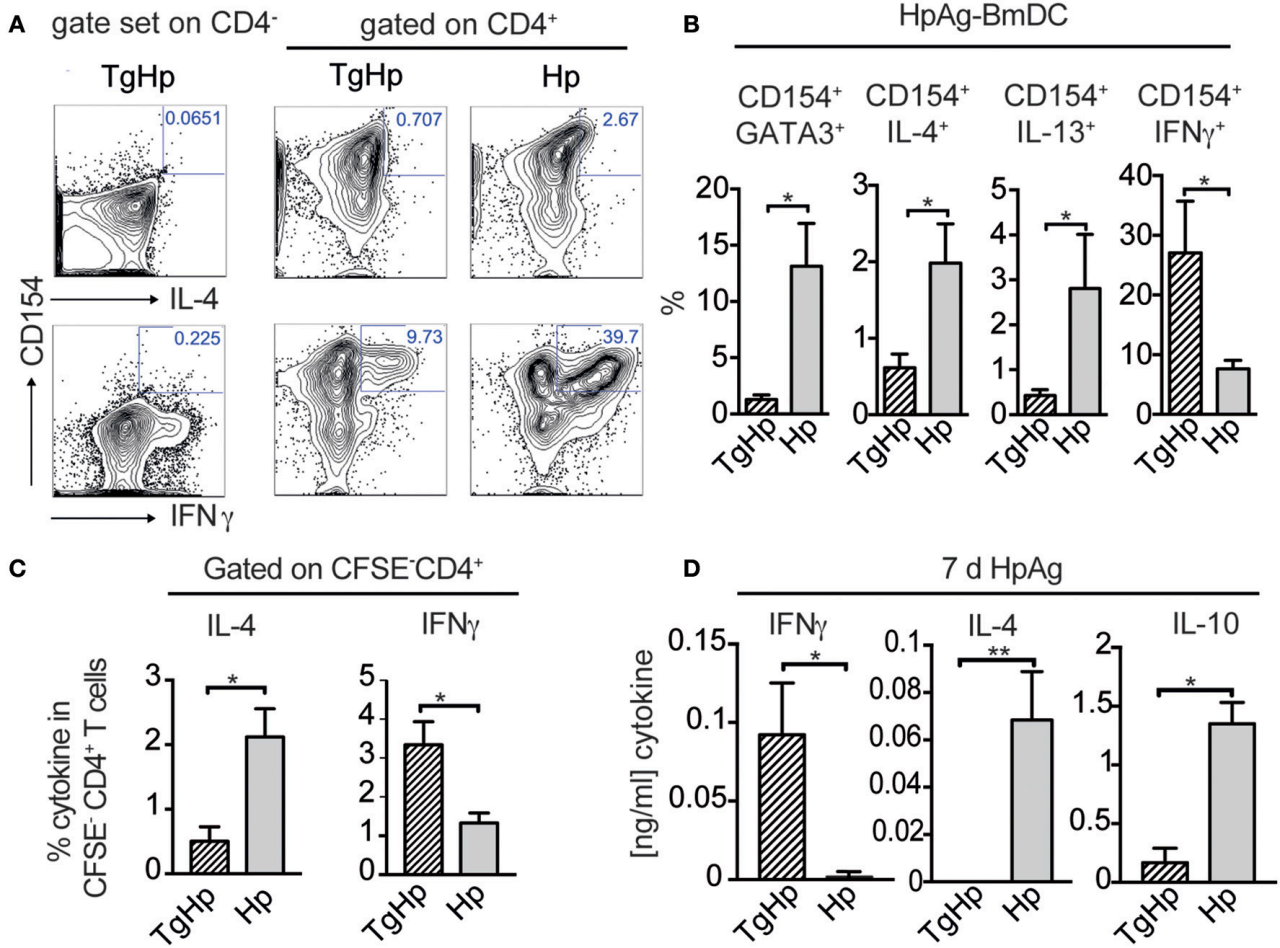
Helminth-reactive CD4^+^ T cells from co-infected mice show enhanced production of IFN-γ. BmDC (1 × 10^5^) were pulsed with 50 μg/ml H. polygyrus antigen (HpAg) and cultured with 1 × 10^6^ splenocytes from single or co-infected animals for 5 h followed by intracellular staining for CD154 and cytokines. Gating strategy is shown in **(A)** whereby the gate for cells coexpressing CD154 and IL-4, IL-13, or IFN-γ was set using CD4^−^ cells. **(B)** Bar graphs showing frequencies of CD154^+^GATA3^+^, CD154^+^IL-4^+^, CD154^+^IL-13^+^, and CD154^+^IFN-γ^+^ in CD4^+^ T cells. Data shown as mean ± SD and representative of two independent experiments with *n* = 4–5. **(C)** CFSE labeled CD4^+^ spleen cells were stimulated for 6 days with 20 μg/ml HpAg. Proliferating spleen cells were gated on (CFSE-CD4+). CD4^+^ cells were re-stimulated with PMA/Ionomycin and stained for intracellular cytokines IL-4, and IFN-γ; data shown as mean ± SD and is representative for two independent experiments *n* = 4. **(D)** Bar graphs show detection of IFN-γ, IL-4 and IL-10 by ELISA from collected supernatant of 3 × 10^5^ splenocytes stimulated with *H. polygyrus* antigen for 6 days; representative of two independent experiments *n* = 4–5, mean ± SD. Statistical analysis was performed using the Mann-Whitney test, ^*^P ≤ 0.05, ^**^P ≤ 0.01.

The lack of the Th2 immune responses in co-infection may suggest that the CD4^+^ T cells are not responding to *H. polygyrus* infection. Thus, we aimed to identify helminth antigen-specific CD4^+^ T cells in co-infected mice. For this we generated bone marrow derived dendritic cells (BmDC) from naïve animals pulsed with *H. polygyrus* antigen (HpAg) and co-cultured them with splenocytes from single and co-infected animals. The antigen-reactive CD4^+^ cells were subsequently detected by the activation marker CD154 (CD40L) (Figure 5A; Frentsch et al., 2005). We observed a reduction in IL-4 and IL-13 in activated CD154^+^CD4^+^ cells in the co-infected group compared to the *H. polygyrus* infected group. Interestingly, despite being unable to produce Th2 cytokines, significant numbers of CD154^+^CD4^+^ T cells from the co-infected group were able to produce IFN-γ in response to HpAg, which was not observed in the *H. polygyrus*-single infected group (Figure 5B). CFSE staining can be used to identify proliferating cells due to halving of CFSE in daughter cells during proliferation. Here, we assessed the proliferation of splenocytes from single and co-infected animals in response to HpAg by gating on CFSE^−^ cells. Interestingly, while CD4^+^ T cells from both groups proliferated based on CFSE-staining, the Th2 cytokine IL-4 was reduced in co-infected animals compared to helminth single infection. On the other hand, IFN-γ production in response to HpAg was increased in co-infected animals (Figure 5C). Additionally, *in vitro* stimulation of splenocytes with HpAg showed similar results. Here, significant amounts of IL-4 and IL-10 could be detected in supernatants from mice infected with *H. polygyrus* only, while IFN-γ was significantly increased in co-infected animals (Figure 5D). Hence, this data supports the observation that helminth-specific CD4^+^ T cells from co-infected animals are unable to commit to the Th2 lineage but produce the Th1 cytokine IFN-γ in response to a helminth infection instead.

## Discussion

In this study a previous low-dose oral infection with *T. gondii* prevented the establishment of local and systemic Th2 responses normally induced by infection with *H. polygyrus*. Neither Th2 cytokines nor the transcription factor GATA3 as well as features triggered by IL-4 (IgG1 antibodies, RELM-ß) or IL-5 (eosinophilia) could be detected in co-infected animals. The observed increase in female worm fecundity in co-infected animals is a likely consequence of a lack in Th2 responses due to the reduced antibody levels and effector molecule RELM-ß, as both were shown to influence *H. polygyrus* fecundity (Owyang et al., 2006; McCoy et al., 2008). Interestingly the Th1 response to *T. gondii* was not diminished in CD4^+^ and CD8^+^ T cells in co-infected animals, since both subsets were able to produce similar amounts of IFN-γ in contrast to other studies, where the helminth infection precedes the protozoan infection (Khan et al., 2008; Marple et al., 2017). This observation is in line with unaltered parasitemia of *T. gondii* during chronic phase of infection. Furthermore, inflammatory monocytes expressing Ly6C infiltrate the brain to control *T. gondii* via the production of pro-inflammatory mediators, such as TNF-α, IL-1-α IL-1-β and nitric oxide synthase (Dunay et al., 2008, 2010; Biswas et al., 2015). In co-infected animals the frequency of inflammatory monocytes was not altered, but the capacity to produce TNF-α in response to LPS was reduced. However, this had no effect on parasitemia of *T. gondii*.

The observation of an apparent reduction in Th2 responses might be due to abolished priming and polarization events that occur at various stages, such as insufficient priming of naïve CD4^+^ helper T cells, altered function of dendritic cells (DC) leading to aberrant polarization, or the local cytokine milieu present at the time of helminth infection. Moreover, a recent study has shown that systemic *T. gondii* infection leads to a long-term defect in the generation and function of naive T lymphocytes (Kugler et al., 2016). However, our findings suggest that in a low-dose infection with *T. gondii*, this effect is not as drastic,since helminth-specific CD4^+^ T cells were shown to proliferate and respond to antigen as seen in Figure 5.

The differentiation between Th1 and Th2 cells requires positive feedback loops and cross-inhibition of other lineages for uniform Th cell differentiation (Mosmann and Coffman, 1989; Paul and Seder, 1994). In addition to this the transcription factor specific for Th1, T-bet, has the ability to suppress the Th2 transcription factor GATA-3. This might provide an explanation for the cross-regulation of cytokines in Th cell differentiation (Hwang et al., 2005). During *T. gondii* infection T-bet can suppress IL-4 and GATA-3 expression, thus, preventing endogenous Th2 cell associated programming (Zhu et al., 2012). In our study, the observed up-regulation of T-bet was in line with the significant reduction of GATA-3 in spleen, siLP and siEL. The reduction in GATA-3 expression followed the absent Th2 cytokine production observed in co-infection with *T. gondii*. Our study is in line with other studies showing suppression in the Th2 responses against helminths, when another pathogen is involved prior or at the same time during infection (Liesenfeld et al., 2004; Lass et al., 2013; Nel et al., 2014). Interestingly, the frequencies of CD4^+^ T-cells from co-infected animals producing either T-bet or IFN-γ are similar to mice infected with *T. gondii* alone or even higher. This suggests that CD4^+^ T cells expressing T-bet and IFN-γ in coinfected animals consist of pre-existing *T. gondii*-specific T cells and *H. polygyrus*-specific T-cells able to produce T-bet and IFN-γ in this coinfection setting.

In general, helminths are shown to actively induce a Th2 program that requires the programming of dendritic cells (DCs) (Steinfelder et al., 2009). DCs from skin LN exposed to *Nippostrongylus brasiliensis* showed transcriptional changes of different DC subsets (Connor et al., 2017). Generally, DCs are pivotal in eliciting Th2 cell responses *in vivo*. A depletion of the CD11c^+^ DCs subset during infection with *S. mansoni* led to an abolished Th2 response and an increase in the production of IFN-γ (Phythian-Adams et al., 2010). This reduced Th2 response was also observed in *H. polygyrus* infection when mice were depleted of CD11c^+^ DCs (Smith et al., 2011). However, during *T. gondii* infection, the induced Th1 immune response is dependent on early IL-12 production by APCs (Scanga et al., 2002). DCs prime naïve T cells, but are also a target of effector cytokines produced by previously polarized effector T cells and innate cells. In our study it is most likely that DCs are affected by the previous and ongoing infection with *T. gondii* and the ensuing cytokine milieu. The presence of IL-12 and IFN-γ at the time point and site of infection as shown in Figure S2 might impact the ability of local DCs to be able to prime naïve T cells for Th2 differentiation. Future studies should investigate the underlying mechanisms and involvement of DCs, such as CD8α^+^ DCs that are a source of IL-12 during *T. gondii* infection (Mashayekhi et al., 2011).

Dendritic cells can also be primed in response to epithelium-derived cytokines known as alarmins. These cytokines are released during epithelial tissue damage (Swamy et al., 2010). Thymic stromal lymphopoietin (TSLP) plays an important role in mounting a Th2 response in *H. polygyrus* infection (Massacand et al., 2009). Also IL-33 is an alarmin and it has been shown that when DCs are treated with IL-33 they polarize CD4 T cells to produce Th2 cytokines (Besnard et al., 2011; Eiwegger and Akdis, 2011). Another tissue derived cytokine; IL-25 has been demonstrated to be involved in Th2 cytokine responses and *N. brasiliensis* expulsion. However, this cytokine has not been described to directly act on DCs (Fallon et al., 2006; Wang et al., 2007). Since early Th2 polarization is dependent on the release of these cytokines that act as alarmins it might be fruitful to investigate these cytokines very early after infection with *H. polygyrus* in previously infected *T. gondii* mice.

Importantly, we show that while helminth-reactive CD4^+^ T cells are unable to produce Th2 cytokines in a co-infection, they still express significant amounts of IFN-γ after restimulation with helminth antigen. We identified IFN-γ producing *H. polygyrus* antigen (HpAg)-reactive T cells in co-infected animals using the activation marker CD154 and a short stimulation protocol (Frentsch et al., 2005; Chattopadhyay et al., 2006). We also saw this in CFSE^−^CD4^+^ T cells that expanded in response to HpAg. In line with our findings, Coomes et al. have shown that a co-infection with *Plasmodium chabaudi* and *H. polygyrus* led to a reduction in Th2 responses. Furthermore, they observed up-regulation of IFN-γ when Th2 cells from *H. polygyrus-*infected mice were adoptively transferred into *Rag1*^−/−^ mice infected with *P. chabaudi*. However, blocking of IL-12 and IFN-γ only partially preserved Th2 immunity in response to *H. polygyrus* (Coomes et al., 2015).

In summary, our data on helminth-antigen specific restimulation of CD4^+^ T cells suggest that naïve CD4^+^ T cells harboring a cognate TCR for helminth antigen fail to commit to the Th2 lineage and are polarized toward a Th1 phenotype in mice previously infected with *Toxoplasma*. This switch in cytokine expression leads to the absence of effector features downstream of the Th2 response and consequently to higher worm fecundity in co-infected animals. Recent studies emphasized the importance of bystander activation and concurrent infections on the outcome of the immune response to unrelated pathogens or vaccines (Reese et al., 2016; Tao and Reese, 2017). In regards to the differences in the development of protective Th2 immunity observed in our study and by others in both mice and humans, it is important to focus on infections not only in a “clean” host but also in the context of individual infection history as well as co-infections.

## Author contributions

NA, SS, SR, TF, and KH performed all the experiments. SH, ID, and SS conceptualized and designed the research. NA, SS, and SH wrote the manuscript. All authors approved the final version of the manuscript.

### Conflict of interest statement

The authors declare that the research was conducted in the absence of any commercial or financial relationships that could be construed as a potential conflict of interest.

## References

[B1] Artis D. Wang M. L. Keilbaugh S. A. He W. Brenes M. Swain G. P.. (2004). RELMbeta/FIZZ2 is a goblet cell-specific immune-effector molecule in the gastrointestinal tract. Proc. Natl. Acad. Sci. U.S.A. 101, 13596–13600. 10.1073/pnas.0404034101 15340149 PMC518800

[B2] Bahia-Oliveira L. M. da Silva J. A. Peixoto-Rangel A. L. Boechat M. S. B. Oliveira A. M. W. A. Massara C. L.. (2009). Host immune response to *Toxoplasma gondii* and *Ascaris lumbricoides* in a highly endemic area: evidence of parasite co-immunomodulation properties influencing the outcome of both infections. Mem. Inst. Oswaldo Cruz 104, 273–280. 10.1590/S0074-02762009000200021 19430653

[B3] Bansemir A. D. Sukhdeo M. V. (1994). The food resource of adult *Heligmosomoides polygyrus* in the small intestine. J. Parasitol. 80, 24–28. 10.2307/3283340 8308654

[B4] Besnard A.-G. Togbe D. Guillou N. Erard F. Quesniaux V. Ryffel B. (2011). IL-33-activated dendritic cells are critical for allergic airway inflammation. Eur. J. Immunol. 41, 1675–1686. 10.1002/eji.201041033 21469105

[B5] Biswas A. Bruder D. Wolf S. A. Jeron A. Mack M. Heimesaat M. M.. (2015). LY6C(high) monocytes control cerebral toxoplasmosis. J. Immunol. 194, 3223–3235. 10.4049/jimmunol.1402037 25710908

[B6] Budischak S. A. Sakamoto K. Megow L. C. Cummings K. R. Urban J. F. Ezenwa V. O. (2015). Resource limitation alters the consequences of co-infection for both hosts and parasites. Int. J. Parasitol. 45, 455–463. 10.1016/j.ijpara.2015.02.005 25812832

[B7] Chattopadhyay P. K. Yu J. Roederer M. (2006). Live-cell assay to detect antigen-specific CD4^+^ T-cell responses by CD154 expression. Nat. Protoc. 1, 1–6. 10.1038/nprot.2006.1 17406204

[B8] Chen C.-C. Louie S. McCormick B. A. Walker W. A. Shi H. N. (2006). Helminth-primed dendritic cells alter the host response to enteric bacterial infection. J. Immunol. 176, 472–483. 10.4049/jimmunol.176.1.472 16365440 PMC4144328

[B9] Chen C.-C. Louie S. McCormick B. Walker W. A. Shi H. N. (2005). Concurrent infection with an intestinal helminth parasite impairs host resistance to enteric *Citrobacter rodentium* and enhances *Citrobacter*-induced colitis in mice. Infect. Immun. 73, 5468–5481. 10.1128/IAI.73.9.5468-5481.2005 16113263 PMC1231118

[B10] Connor L. M. Tang S.-C. Cognard E. Ochiai S. Hilligan K. L. Old S. I.. (2017). Th2 responses are primed by skin dendritic cells with distinct transcriptional profiles. J. Exp. Med. 214, 125–142. 10.1084/jem.20160470 27913566 PMC5206495

[B11] Coomes S. M. Pelly V. S. Wilson M. S. (2013). Plasticity within the αβ^+^CD4^+^ T-cell lineage: when, how and what for? Open Biol. 3:120157. 10.1098/rsob.120157 23345540 PMC3603458

[B12] Coomes S. M. Pelly V. S. Kannan Y. Okoye I. S. Czieso S. Entwistle L. J.. (2015). IFN-γ and IL-12 restrict Th2 responses during Helminth/*Plasmodium* co-infection and promote IFN-γ from Th2 cells. PLoS Pathog. 11:e1004994. 10.1371/journal.ppat.1004994 26147567 PMC4493106

[B13] Dias A. T. de Castro S. B. R. Alves C. C. S. Rezende A. B. Rodrigues M. F. Machado R. R. P.. (2011). Lower production of IL-17A and increased susceptibility to *Mycobacterium bovis* in mice coinfected with *Strongyloides venezuelensis*. Mem. Inst. Oswaldo Cruz 106, 617–619. 10.1590/S0074-02762011000500015 21894384

[B14] du Plessis N. Kleynhans L. Thiart L. van Helden P. D. Brombacher F. Horsnell W. G. C.. (2013). Acute helminth infection enhances early macrophage mediated control of mycobacterial infection. Mucosal Immunol. 6, 931–941. 10.1038/mi.2012.131 23250274

[B15] Dubey J. P. (2008). The history of *Toxoplasma gondii*–the first 100 years. J. Eukaryot. Microbiol. 55, 467–475. 10.1111/j.1550-7408.2008.00345.x 19120791

[B16] Dunay I. R. Damatta R. A. Fux B. Presti R. Greco S. Colonna M.. (2008). Gr1^+^ inflammatory monocytes are required for mucosal resistance to the pathogen *Toxoplasma gondii*. Immunity 29, 306–317. 10.1016/j.immuni.2008.05.019 18691912 PMC2605393

[B17] Dunay I. R. Fuchs A. Sibley L. D. (2010). Inflammatory monocytes but not neutrophils are necessary to control infection with *Toxoplasma gondii* in mice. Infect. Immun. 78, 1564–1570. 10.1128/IAI.00472-09 20145099 PMC2849397

[B18] Eiwegger T. Akdis C. A. (2011). IL-33 links tissue cells, dendritic cells and Th2 cell development in a mouse model of asthma. Eur. J. Immunol. 41, 1535–1538. 10.1002/eji.201141668 21618506

[B19] Ely K. H. Kasper L. H. Khan I. A. (1999). Augmentation of the CD8+ T cell response by IFN-gamma in IL-12-deficient mice during *Toxoplasma gondii* infection. J. Immunol. 162, 5449–5454. 10228024

[B20] Fallon P. G. Ballantyne S. J. Mangan N. E. Barlow J. L. Dasvarma A. Hewett D. R.. (2006). Identification of an interleukin (IL)-25-dependent cell population that provides IL-4, IL-5, and IL-13 at the onset of helminth expulsion. J. Exp. Med. 203, 1105–1116. 10.1084/jem.20051615 16606668 PMC2118283

[B21] Frantz F. G. Rosada R. S. Peres-Buzalaf C. Perusso F. R. T. Rodrigues V. Ramos S. G.. (2010). Helminth coinfection does not affect therapeutic effect of a DNA vaccine in mice harboring tuberculosis. PLoS Negl. Trop. Dis. 4:e700. 10.1371/journal.pntd.0000700 20544012 PMC2882318

[B22] Frentsch M. Arbach O. Kirchhoff D. Moewes B. Worm M. Rothe M.. (2005). Direct access to CD4^+^ T cells specific for defined antigens according to CD154 expression. Nat. Med. 11, 1118–1124. 10.1038/nm1292 16186818

[B23] Gazzinelli R. T. Wysocka M. Hayashi S. Denkers E. Y. Hieny S. Caspar P.. (1994). Parasite-induced IL-12 stimulates early IFN-gamma synthesis and resistance during acute infection with *Toxoplasma gondii*. J. Immunol. 153, 2533–2543. 7915739

[B24] Gondorf F. Berbudi A. Buerfent B. C. Ajendra J. Bloemker D. Specht S.. (2015). Chronic filarial infection provides protection against bacterial sepsis by functionally reprogramming macrophages. PLoS Pathog. 11:e1004616. 10.1371/journal.ppat.1004616 25611587 PMC4303312

[B25] Gordon S. Martinez F. O. (2010). Alternative activation of macrophages: mechanism and functions. Immunity 32, 593–604. 10.1016/j.immuni.2010.05.007 20510870

[B26] Graham A. L. Lamb T. J. Read A. F. Allen J. E. (2005). Malaria-filaria coinfection in mice makes malarial disease more severe unless filarial infection achieves patency. J. Infect. Dis. 191, 410–421. 10.1086/426871 15633101

[B27] Grogan J. L. Mohrs M. Harmon B. Lacy D. A. Sedat J. W. Locksley R. M. (2001). Early transcription and silencing of cytokine genes underlie polarization of T helper cell subsets. Immunity 14, 205–215. 10.1016/S1074-7613(01)00103-0 11290331

[B28] Heimesaat M. M. Dunay I. R. Schulze S. Fischer A. Grundmann U. Alutis M.. (2014). Pituitary adenylate cyclase-activating polypeptide ameliorates experimental acute ileitis and extra-intestinal sequelae. PLoS ONE 9:e108389. 10.1371/journal.pone.0108389 25238233 PMC4169633

[B29] Horton J. (2003). Human gastrointestinal helminth infections: are they now neglected diseases? Trends Parasitol. 19, 527–531. 10.1016/j.pt.2003.09.007 14580965

[B30] Hotez P. J. Kamath A. (2009). Neglected tropical diseases in sub-saharan Africa: review of their prevalence, distribution, and disease burden. PLoS Negl. Trop. Dis. 3:e412. 10.1371/journal.pntd.0000412 19707588 PMC2727001

[B31] Ho-Yen D. O. Joss A. W. L. (1992). Human Toxoplasmosis, 1st Edn. Oxford; New York, NY: Oxford University Press.

[B32] Hussaarts L. van der Vlugt L. E. P. M. Yazdanbakhsh M. Smits H. H. (2011). Regulatory B-cell induction by helminths: implications for allergic disease. J. Allergy Clin. Immunol. 128, 733–739. 10.1016/j.jaci.2011.05.012 21684587

[B33] Hwang E. S. Szabo S. J. Schwartzberg P. L. Glimcher L. H. (2005). T helper cell fate specified by kinase-mediated interaction of T-bet with GATA-3. Science 307, 430–433. 10.1126/science.1103336 15662016

[B34] Khan I. A. Hakak R. Eberle K. Sayles P. Weiss L. M. Urban J. F. (2008). Coinfection with *Heligmosomoides polygyrus* fails to establish CD8^+^ T-cell immunity against *Toxoplasma gondii*. Infect. Immun. 76, 1305–1313. 10.1128/IAI.01236-07 18195022 PMC2258819

[B35] Klose C. S. N. Flach M. Möhle L. Rogell L. Hoyler T. Ebert K.. (2014). Differentiation of type 1 ILCs from a common progenitor to all helper-like innate lymphoid cell lineages. Cell 157, 340–356. 10.1016/j.cell.2014.03.030 24725403

[B36] Kolbaum J. Eschbach M.-L. Steeg C. Jacobs T. Fleischer B. Breloer M. (2012). Efficient control of *Plasmodium yoelii* infection in BALB/c and C57BL/6 mice with pre-existing *Strongyloides ratti* infection. Parasite Immunol. 34, 388–393. 10.1111/j.1365-3024.2012.01369.x 22554071

[B37] Kugler D. G. Flomerfelt F. A. Costa D. L. Laky K. Kamenyeva O. Mittelstadt P. R.. (2016). Systemic toxoplasma infection triggers a long-term defect in the generation and function of naive T lymphocytes. J. Exp. Med. 213, 3041–3056. 10.1084/jem.20151636 27849554 PMC5154934

[B38] Lass S. Hudson P. J. Thakar J. Saric J. Harvill E. Albert R.. (2013). Generating super-shedders: co-infection increases bacterial load and egg production of a gastrointestinal helminth. J. R. Soc. Interface 10:20120588. 10.1098/rsif.2012.0588 23256186 PMC3565725

[B39] Liesenfeld O. Dunay I. R. Erb K. J. (2004). Infection with *Toxoplasma gondii* reduces established and developing Th2 responses induced by *Nippostrongylus brasiliensis* infection. Infect. Immun. 72, 3812–3822. 10.1128/IAI.72.7.3812-3822.2004 15213122 PMC427426

[B40] Luft B. J. Brooks R. G. Conley F. K. McCabe R. E. Remington J. S. (1984). Toxoplasmic encephalitis in patients with acquired immune deficiency syndrome. JAMA 252, 913–917. 10.1001/jama.1984.03350070031018 6748191

[B41] Maaz D. Rausch S. Richter D. Krücken J. Kühl A. A. Demeler J.. (2016). Susceptibility to ticks and Lyme disease spirochetes is not affected in mice co-infected with nematodes. Infect. Immun. 84, 1274–1286. 10.1128/IAI.01309-15 26883594 PMC4862734

[B42] Marple A. Wu W. Shah S. Zhao Y. Du P. Gause W. C.. (2017). Cutting edge: helminth coinfection blocks effector differentiation of CD8 T cells through alternate host Th2- and IL-10–mediated responses. J. Immunol. 198, 634–639. 10.4049/jimmunol.1601741 27956529 PMC5225035

[B43] Mashayekhi M. Sandau M. M. Dunay I. R. Frickel E. M. Khan A. (2011). CD8α^+^ dendritic cells are the critical source of interleukin-12 that controls acute infection by *Toxoplasma gondii* Tachyzoites. Immunity 35, 249–259. 10.1016/j.immuni.2011.08.008 21867928 PMC3171793

[B44] Massacand J. C. Stettler R. C. Meier R. Humphreys N. E. Grencis R. K. Marsland B. J.. (2009). Helminth products bypass the need for TSLP in Th2 immune responses by directly modulating dendritic cell function. Proc. Natl. Acad. Sci. U.S.A. 106, 13968–13973. 10.1073/pnas.0906367106 19666528 PMC2729004

[B45] McCoy K. D. Stoel M. Stettler R. Merky P. Fink K. Senn B. M.. (2008). Polyclonal and specific antibodies mediate protective immunity against enteric helminth infection. Cell Host Microbe 4, 362–373. 10.1016/j.chom.2008.08.014 18854240

[B46] McSorley H. J. Maizels R. M. (2012). Helminth infections and host immune regulation. Clin. Microbiol. Rev. 25, 585–608. 10.1128/CMR.05040-11 23034321 PMC3485755

[B47] Miller C. M. D. Smith N. C. Ikin R. J. Boulter N. R. Dalton J. P. Donnelly S. (2009). Immunological interactions between 2 common pathogens, Th1-inducing protozoan *Toxoplasma gondii* and the Th2-inducing helminth *Fasciola hepatica*. PLoS ONE 4:e5692. 10.1371/journal.pone.0005692 19478853 PMC2682559

[B48] Möhle L. Israel N. Paarmann K. Krohn M. Pietkiewicz S. Müller A.. (2016). Chronic *Toxoplasma gondii* infection enhances β-amyloid phagocytosis and clearance by recruited monocytes. Acta Neuropathol. Commun. 4:25. 10.1186/s40478-016-0293-8 26984535 PMC4793516

[B49] Montoya J. G. Liesenfeld O. (2004). Toxoplasmosis. Lancet Lond. Engl. 363, 1965–1976. 10.1016/S0140-6736(04)16412-X 15194258

[B50] Mosmann T. R. Coffman R. L. (1989). TH1 and TH2 cells: different patterns of lymphokine secretion lead to different functional properties. Annu. Rev. Immunol. 7, 145–173. 10.1146/annurev.iy.07.040189.001045 2523712

[B51] Murphy E. Shibuya K. Hosken N. Openshaw P. Maino V. Davis K.. (1996). Reversibility of T helper 1 and 2 populations is lost after long-term stimulation. J. Exp. Med. 183, 901–913. 8642294 10.1084/jem.183.3.901 PMC2192360

[B52] Murphy K. M. Stockinger B. (2010). Effector T cell plasticity: flexibility in the face of changing circumstances. Nat. Immunol. 11, 674–680. 10.1038/ni.1899 20644573 PMC3249647

[B53] Nel H. J. du Plessis N. Kleynhans L. Loxton A. G. van Helden P. D. Walzl G. (2014). *Mycobacterium bovis* BCG infection severely delays *Trichuris muris* expulsion and co-infection suppresses immune responsiveness to both pathogens. BMC Microbiol. 14:9. 10.1186/1471-2180-14-9 24433309 PMC3898725

[B54] Noland G. S. Urban J. F. Fried B. Kumar N. (2008). Counter-regulatory anti-parasite cytokine responses during concurrent *Plasmodium yoelii* and intestinal helminth infections in mice. Exp. Parasitol. 119, 272–278. 10.1016/j.exppara.2008.02.009 18396282 PMC2441905

[B55] O'Garra A. Gabryšová L. Spits H. (2011). Quantitative events determine the differentiation and function of helper T cells. Nat. Immunol. 12, 288–294. 10.1038/ni.2003 21423225

[B56] Obieglo K. Feng X. Bollampalli V. P. Dellacasa-Lindberg I. Classon C. Österblad M. (2016). Chronic gastrointestinal nematode infection mutes immune responses to Mycobacterial infection distal to the gut. J. Immunol. 196, 2262–2271. 10.4049/jimmunol.1500970 26819205 PMC4760231

[B57] Osborne L. C. Monticelli L. A. Nice T. J. Sutherland T. E. Siracusa M. C. Hepworth M. R.. (2014). Coinfection. virus-helminth coinfection reveals a microbiota-independent mechanism of immunomodulation. Science 345, 578–582. 10.1126/science.1256942 25082704 PMC4548887

[B58] Owyang A. M. Zaph C. Wilson E. H. Guild K. J. McClanahan T. Miller H. R. P.. (2006). Interleukin 25 regulates type 2 cytokine-dependent immunity and limits chronic inflammation in the gastrointestinal tract. J. Exp. Med. 203, 843–849. 10.1084/jem.20051496 16606667 PMC1800834

[B59] Pappas G. Roussos N. Falagas M. E. (2009). Toxoplasmosis snapshots: global status of T*oxoplasma gondii* seroprevalence and implications for pregnancy and congenital toxoplasmosis. Int. J. Parasitol. 39, 1385–1394. 10.1016/j.ijpara.2009.04.003 19433092

[B60] Parlog A. Schlüter D. Dunay I. R. (2015). *Toxoplasma gondii*-induced neuronal alterations. Parasite Immunol. 37, 159–170. 10.1111/pim.12157 25376390

[B61] Paul W. E. Seder R. A. (1994). Lymphocyte responses and cytokines. Cell 76, 241–251. 10.1016/0092-8674(94)90332-8 7904900

[B62] Peine M. Rausch S. Helmstetter C. Fröhlich A. Hegazy A. N. Kühl A. A.. (2013). Stable T-bet^+^GATA-3^+^ Th1/Th2 hybrid cells arise *in vivo*, can develop directly from naive precursors, and limit immunopathologic inflammation. PLoS Biol. 11:e1001633. 10.1371/journal.pbio.1001633 23976880 PMC3747991

[B63] Phythian-Adams A. T. Cook P. C. Lundie R. J. Jones L. H. Smith K. A. Barr T. A.. (2010). CD11c depletion severely disrupts Th2 induction and development *in vivo*. J. Exp. Med. 207, 2089–2096. 10.1084/jem.20100734 20819926 PMC2947067

[B64] Potian J. A. Rafi W. Bhatt K. McBride A. Gause W. C. Salgame P. (2011). Preexisting helminth infection induces inhibition of innate pulmonary anti-tuberculosis defense by engaging the IL-4 receptor pathway. J. Exp. Med. 208, 1863–1874. 10.1084/jem.20091473 21825018 PMC3171086

[B65] Rafi W. Bhatt K. Gause W. C. Salgame P. (2015). Neither primary nor memory immunity to *Mycobacterium tuberculosis* infection is compromised in mice with chronic enteric helminth infection. Infect. Immun. 83, 1217–1223. 10.1128/IAI.03004-14 25605766 PMC4333454

[B66] Rausch S. Huehn J. Kirchhoff D. Rzepecka J. Schnoeller C. Pillai S.. (2008). Functional analysis of effector and regulatory T cells in a parasitic nematode infection. Infect. Immun. 76, 1908–1919. 10.1128/IAI.01233-07 18316386 PMC2346705

[B67] Reese T. A. Bi K. Kambal A. Filali-Mouhim A. Beura L. K. Bürger M. C.. (2016). Sequential Infection with common pathogens promotes human-like immune gene expression and altered vaccine response. Cell Host Microbe 19, 713–719. 10.1016/j.chom.2016.04.003 27107939 PMC4896745

[B68] Reynolds L. A. Filbey K. J. Maizels R. M. (2012). Immunity to the model intestinal helminth parasite *Heligmosomoides polygyrus*. Semin. Immunopathol. 34, 829–846. 10.1007/s00281-012-0347-3 23053394 PMC3496515

[B69] Rousseau D. Le Fichoux Y. Stien X. Suffia I. Ferrua B. Kubar J. (1997). Progression of visceral leishmaniasis due to Leishmania infantum in BALB/c mice is markedly slowed by prior infection with *Trichinella spiralis*. Infect. Immun. 65, 4978–4983. 9393785 10.1128/iai.65.12.4978-4983.1997 PMC175718

[B70] Scanga C. A. Aliberti J. Jankovic D. Tilloy F. Bennouna S. Denkers E. Y.. (2002). Cutting edge: MyD88 is required for resistance to *Toxoplasma gondii* Infection and regulates parasite-induced IL-12 production by dendritic cells. J. Immunol. 168, 5997–6001. 10.4049/jimmunol.168.12.5997 12055206

[B71] Smith K. A. Hochweller K. Hämmerling G. J. Boon L. MacDonald A. S. Maizels R. M. (2011). Chronic helminth infection promotes immune regulation *in vivo* through dominance of CD11cloCD103- dendritic cells. J. Immunol. 186, 7098–7109. 10.4049/jimmunol.1003636 21576507 PMC4794626

[B72] Steinfelder S. Andersen J. F. Cannons J. L. Feng C. G. Joshi M. Dwyer D.. (2009). The major component in schistosome eggs responsible for conditioning dendritic cells for Th2 polarization is a T2 ribonuclease (omega-1). J. Exp. Med. 206, 1681–1690. 10.1084/jem.20082462 19635859 PMC2722182

[B73] Steinfelder S. O'Regan N. L. Hartmann S. (2016). Diplomatic assistance: can helminth-modulated macrophages act as treatment for inflammatory disease? PLoS Pathog. 12:e1005480. 10.1371/journal.ppat.1005480 27101372 PMC4839649

[B74] Sturge C. R. Benson A. Raetz M. Wilhelm C. L. Mirpuri J. Vitetta E. S.. (2013). TLR-independent neutrophil-derived IFN-γ is important for host resistance to intracellular pathogens. Proc. Natl. Acad. Sci. U.S.A. 110, 10711–10716. 10.1073/pnas.1307868110 23754402 PMC3696766

[B75] Su L. Qi Y. Zhang M. Weng M. Zhang X. Su C.. (2014a). Development of fatal intestinal inflammation in MyD88 deficient mice co-infected with helminth and bacterial enteropathogens. PLoS Negl. Trop. Dis. 8:e2987. 10.1371/journal.pntd.0002987 25010669 PMC4091940

[B76] Su L. Su C. Qi Y. Yang G. Zhang M. Cherayil B. J.. (2014b). Coinfection with an intestinal helminth impairs host innate immunity against *Salmonella enterica* serovar Typhimurium and exacerbates intestinal inflammation in mice. Infect. Immun. 82, 3855–3866. 10.1128/IAI.02023-14 24980971 PMC4187801

[B77] Su Z. Segura M. Morgan K. Loredo-Osti J. C. Stevenson M. M. (2005). Impairment of protective immunity to blood-stage malaria by concurrent nematode infection. Infect. Immun. 73, 3531–3539. 10.1128/IAI.73.6.3531-3539.2005 15908382 PMC1111846

[B78] Swamy M. Jamora C. Havran W. Hayday A. (2010). Epithelial decision makers: in search of the “epimmunome.” Nat. Immunol. 11, 656–665. 10.1038/ni.1905 20644571 PMC2950874

[B79] Szabo S. J. Kim S. T. Costa G. L. Zhang X. Fathman C. G. Glimcher L. H. (2000). A novel transcription factor, T-bet, directs Th1 lineage commitment. Cell 100, 655–669. 10.1016/S0092-8674(00)80702-3 10761931

[B80] Tao L. Reese T. A. (2017). Making mouse models that reflect human immune responses. Trends Immunol. 38, 181–193. 10.1016/j.it.2016.12.007 28161189

[B81] Taylor M. D. van der Werf N. Maizels R. M. (2012). T cells in helminth infection: the regulators and the regulated. Trends Immunol. 33, 181–189. 10.1016/j.it.2012.01.001 22398370

[B82] Wang Y.-H. Angkasekwinai P. Lu N. Voo K. S. Arima K. Hanabuchi S.. (2007). IL-25 augments type 2 immune responses by enhancing the expansion and functions of TSLP-DC-activated Th2 memory cells. J. Exp. Med. 204, 1837–1847. 10.1084/jem.20070406 17635955 PMC2118667

[B83] Weng M. Huntley D. Huang I.-F. Foye-Jackson O. Wang L. Sarkissian A.. (2007). Alternatively activated macrophages in intestinal helminth infection: effects on concurrent bacterial colitis. J. Immunol. 179, 4721–4731. 10.4049/jimmunol.179.7.4721 17878371 PMC3208515

[B84] WHO (2017). Soil-Transmitted Helminth Infections. Available online at: http://www.who.int/mediacentre/factsheets/fs366/en/

[B85] Wohlfert E. A. Grainger J. R. Bouladoux N. Konkel J. E. Oldenhove G. Ribeiro C. H.. (2011). GATA3 controls Foxp3^+^ regulatory T cell fate during inflammation in mice. J. Clin. Invest. 121, 4503–4515. 10.1172/JCI57456 21965331 PMC3204837

[B86] Yazdanbakhsh M. van den Biggelaar A. Maizels R. M. (2001). Th2 responses without atopy: immunoregulation in chronic helminth infections and reduced allergic disease. Trends Immunol. 22, 372–377. 10.1016/S1471-4906(01)01958-5 11429321

[B87] Zheng W. Flavell R. A. (1997). The transcription factor GATA-3 is necessary and sufficient for Th2 cytokine gene expression in CD4 T cells. Cell 89, 587–596. 10.1016/S0092-8674(00)80240-8 9160750

[B88] Zhu J. Jankovic D. Oler A. J. Wei G. Sharma S. Hu G.. (2012). The transcription factor T-bet is induced by multiple pathways and prevents an endogenous Th2 cell program during Th1 cell responses. Immunity 37, 660–673. 10.1016/j.immuni.2012.09.007 23041064 PMC3717271

