# Supplementary figures and images for: Toxoplasma Co-infection Prevents Th2 Differentiation and Leads to a Helminth-Specific Th1 Response
Source: Front Cell Infect Microbiol. 2017 Jul 25;7:341. doi: 10.3389/fcimb.2017.00341 (PMC5524676; doi:10.3389/fcimb.2017.00341)

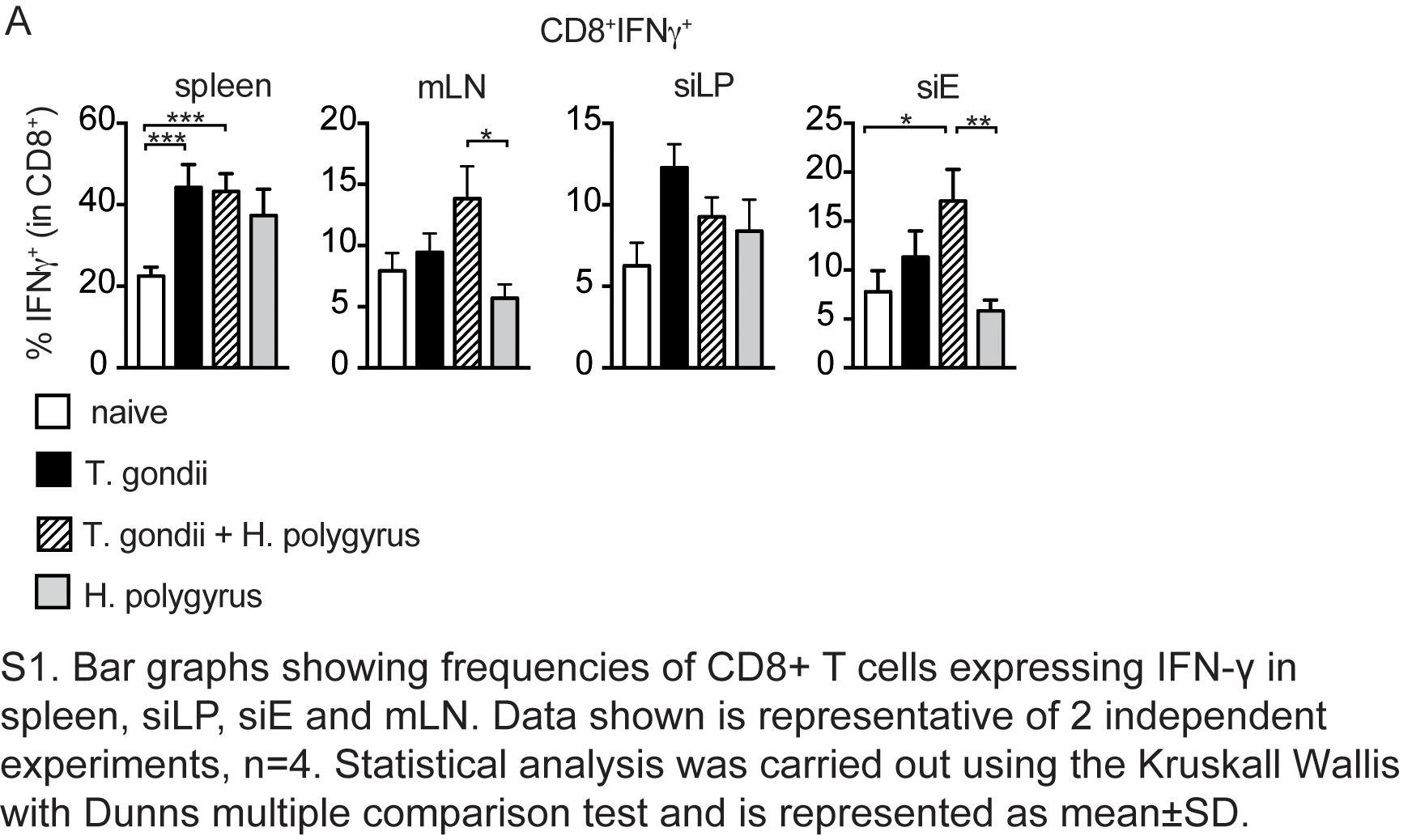

Supplement: Supplementary file 1 [file Image1.tif]
