# Supplementary material for: Toxoplasma Co-infection Prevents Th2 Differentiation and Leads to a Helminth-Specific Th1 Response
Source: Front Cell Infect Microbiol. 2017 Jul 25;7:341. doi: 10.3389/fcimb.2017.00341 (PMC5524676; doi:10.3389/fcimb.2017.00341)
Supplement: Supplementary file 2 [file Image2.PDF]

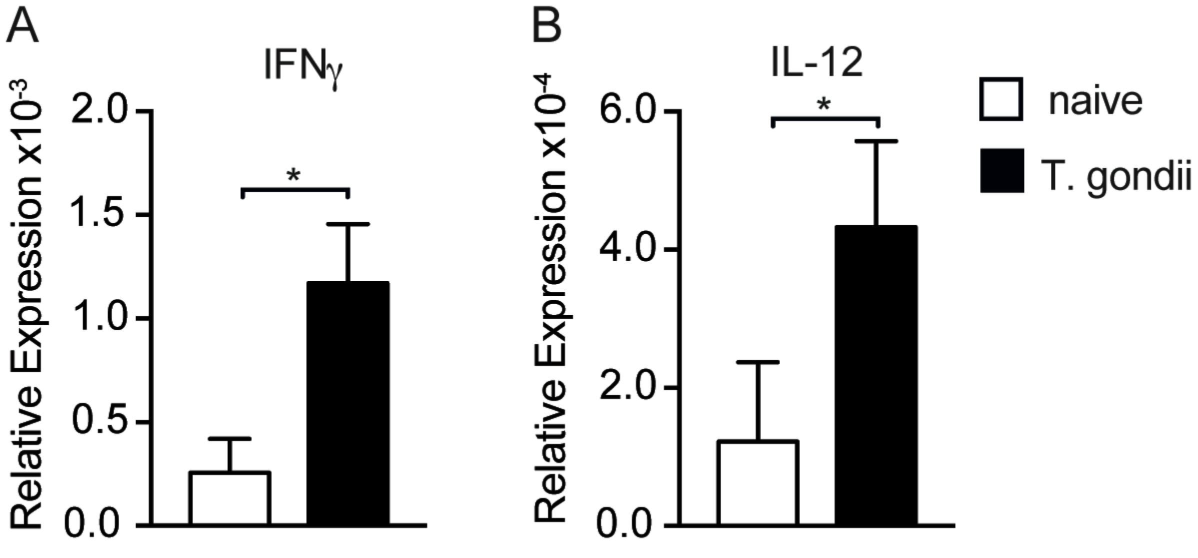

S2. Th1 polarizing environment present in the small intestinal milieu prior to *H. polygyrus* infection

Mice were infected with *T. gondii* and sacrificed on day 14 , A) Relative Gene Expression of IFN-γ (A) and IL-12 (B). Both normalized to β-actin. Data shows mean±SEM. Pooled data from 2 independent experiments with A) n=8-10 and B) n= 7-8 animals each. Statistical analysis was done using the Mann-Whitney test, * p≤ 0,05.
